# Domain adaptation for segmentation of critical structures for prostate cancer therapy

**DOI:** 10.1038/s41598-021-90294-4

**Published:** 2021-06-01

**Authors:** Anneke Meyer, Alireza Mehrtash, Marko Rak, Oleksii Bashkanov, Bjoern Langbein, Alireza Ziaei, Adam S. Kibel, Clare M. Tempany, Christian Hansen, Junichi Tokuda

**Affiliations:** 1 Department of Simulation and Graphics and Research Campus STIMULATE, University of Magdeburg, Magdeburg, Germany; 2 Department of Radiology, Brigham and Women’s Hospital, Harvard Medical School, Boston, MA USA; 3 Division of Urology, Department of Surgery, Brigham and Women’s Hospital, Harvard Medical School, Boston, MA USA

**Keywords:** Prostate, Urethra, Computer science

## Abstract

Preoperative assessment of the proximity of critical structures to the tumors is crucial in avoiding unnecessary damage during prostate cancer treatment. A patient-specific 3D anatomical model of those structures, namely the neurovascular bundles (NVB) and the external urethral sphincters (EUS), can enable physicians to perform such assessments intuitively. As a crucial step to generate a patient-specific anatomical model from preoperative MRI in a clinical routine, we propose a multi-class automatic segmentation based on an anisotropic convolutional network. Our specific challenge is to train the network model on a unique source dataset only available at a single clinical site and deploy it to another target site without sharing the original images or labels. As network models trained on data from a single source suffer from quality loss due to the domain shift, we propose a semi-supervised domain adaptation (DA) method to refine the model’s performance in the target domain. Our DA method combines transfer learning and uncertainty guided self-learning based on deep ensembles. Experiments on the segmentation of the prostate, NVB, and EUS, show significant performance gain with the combination of those techniques compared to pure TL and the combination of TL with simple self-learning (${p}<0.005$ for all structures using a Wilcoxon’s signed-rank test). Results on a different task and data (Pancreas CT segmentation) demonstrate our method’s generic application capabilities. Our method has the advantage that it does not require any further data from the source domain, unlike the majority of recent domain adaptation strategies. This makes our method suitable for clinical applications, where the sharing of patient data is restricted.

## Introduction

Prostate cancer (PCa) is the most common cancer among men and one of the leading causes of cancer death in the United States and other developed countries^1^. Radical prostatectomy is commonly performed as a primary treatment option for PCa, which removes the entire prostate gland regardless of the location of the lesion. Despite their oncologic effectiveness, increasing use of radical treatments among low- and intermediate-risk patients has raised a concern about overtreatment and unnecessary risk of complications^2,3^. Studies have shown that preservation of the neurovascular bundles (NVB) and the external urethral sphincter (EUS) are associated with improved postoperative recovery from impotence and incontinence^4,5^.

With the widespread use of advanced MRI techniques and robot-assisted laparoscopic prostatectomy (RALP), it has become possible to evaluate the involvement of these critical structures in the tumor prior to surgery and spare them to reduce the risk of complications and recovery time^5,6^.

**Figure 1:**
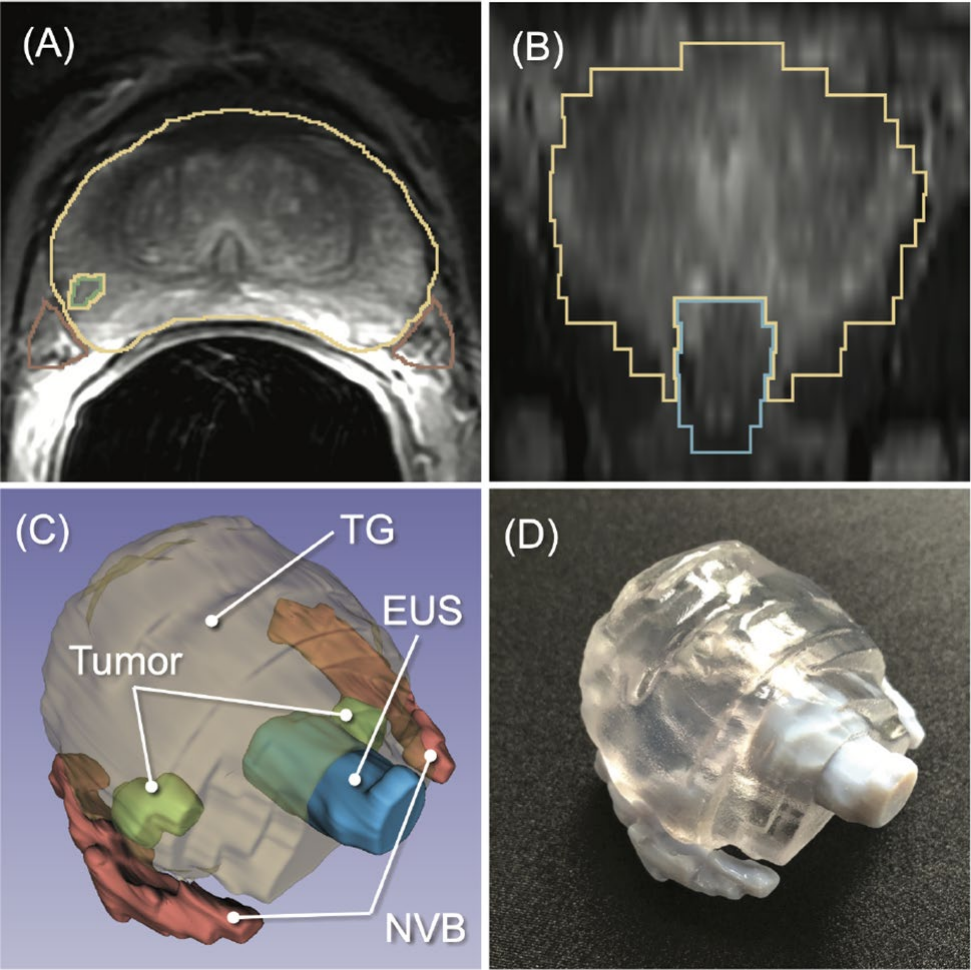
An example application of 3D segmentation of the prostate and adjacent structures for surgical planning. The prostate gland, neurovascular bundle (NVB), external urethral sphincter (EUS), and tumor are manually segmented on the preoperative T2-weighted MRI (**A**, **B**) by a radiologist, and then converted to a 3D surface model (**C**). The model can also be 3D-printed (**D**) for surgical planning, and preoperative communication with the patient.

To facilitate decision-making based on preoperative MRI, researchers have been investigating the impact of patient-specific 3D models^7^. Those models typically include the prostate gland, tumor, NVB, and other surrounding structures, and are presented on a computer display or as a 3D printed model (Fig. 1). Compared to reviewing raw MRI and text reports written by radiologists, the 3D model allows understanding the proximity of the tumor to the critical structures more intuitively. Therefore, they can serve as a tool for surgeons to decide whether to spare the critical structures, as well as for patient information. Despite the growing clinical interest and the availability of 3D visualization software and/or 3D printing technologies, patient-specific 3D models are not routinely used in part due to the lack of robust automatic segmentation of the relevant anatomical structures on preoperative MRI.

There have been several efforts to automatically segment the prostate and tumor using deep learning (DL)^8^. However, these techniques have not fully addressed the clinical need due to several issues. First, the prior studies are focused primarily on either the prostate gland or the tumor and have not included structures relevant to surgical planning, such as the NVB and EUS, partly due to the limited availability of training data that contain expert segmentation of those structures. Second, DL models trained for a specific dataset (*source data*) often do not perform well on a second independent dataset (*target data*) due to the large domain shift (i.e., differences in types of coils, field strength, and MRI parameters). This second issue is particularly critical when the expert segmentation is only available for a small portion of the data. Third, even though it is possible to transfer a model from one dataset to another using *domain adaptation* (DA) techniques^9^, the majority of those techniques require that both source and target data are available. This requirement often becomes a burden, when the model is deployed among multiple institutions while the access to the source data is limited due to the privacy concern.

Therefore, methods that relax the requirement of source data need to be explored. A trained model is less restrictive and easier to share; compared to data from the source domain. Several deployment services exist, that allow sharing the model architecture and weights without the training data for further reuse^10,11^. The concept of federated learning^12^ also exploits the fact that DL models are easier to share than their training data.

This study aims to make automatic segmentation more clinically applicable as a tool to aid the surgical planning process. Specifically, we propose the combination of transfer learning and semi-supervised learning for DA. While both learning techniques have been successfully applied to reduce the amount of labeled data, to the best of our knowledge, they have not been combined for the DA in medical image segmentation yet. In this study, we demonstrated: (1) automatic segmentation of structures relevant to surgical planning, including total gland, NVB, and EUS; and (2) a new DA technique to adapt a convolutional neural network (CNN) model trained on our source dataset to another target dataset acquired at a different institution, with only the source model and no source data available.

## Related work

### Segmentation of prostate and substructures

A variety of MRI prostate segmentation algorithms have been proposed^13^. The majority of recent publications involve CNNs based on the U-Net either in its 2D^14^ or 3D variant^15^. In the context of prostate segmentation, the CNNs have been extended by adapting deep supervision^16–18^ and a multi-planar input^19^ and multi-stream architecture^20^. More recently, residual as well as long and short connections between the layers^17,21–23^ are used to improve performance. Dense connections that enhance feature reuse and propagation have also been shown to improve performance^24–29^. Furthermore, the segmentation can be formulated as a regression task^30^. The authors combined a 3D shape model with a convolutional regression network, where the network is used to obtain the distance from the surface mesh to the corresponding boundary point of the prostate in the image.

In contrast to prostate gland segmentation, less research focused on its internal and adjacent structures. NVB has only been segmented manually on MRI for registration of MR and transrectal ultrasound (TRUS) images^31^. A recent study addresses the segmentation of the distal prostatic urethra in a multi-class segmentation with the zonal anatomy of the prostate^32^. Another study used radiomics features to segment the peripheral zone and the prostatic urethra^33^. No research has been carried out on the automatic segmentation of the EUS that we are aware of currently.

### Domain adaptation and generalization

Deep learning models often fail to achieve robust segmentation in a different domain, making it difficult to be deployed in a wide variety of clinical settings. This is particularly true for studies that require highly-specialized labeled data that is only available in small portions. Several strategies have been used to overcome this challenge, including DA^34^ and *domain generalization* (DG). In the following, we will denote images from the source domain as $X_S$ and images from the target domain as $X_T$. Similarly, we denote labels from the source domain as $Y_s$ and from the target domain as $Y_T$.

DG is a strategy to improve the robustness of neural networks on unseen domains. In contrast to the DA techniques, DG does not require any data from the target domain. Instead, it trains a robust model on a large amount of source data. Intensive data augmentation of $X_S$ and $Y_S$ has been shown to improve the generalization capacity of the network with stacked augmentation transforms^35^. For prostate segmentation, a performance close to the state-of-the-art fully-supervised methods on the target domain was achieved when data augmentation was applied to a large source set ($|(X_S, Y_S)| > 450$). Another DG method applied shape-aware meta-learning to prostate segmentation with promising results compared to other DG techniques^36^—with the downside, data from multiple source domains need to be available.

Compared to DG, more recent works have been published in the field of DA. DA is the strategy to transfer the source model to the target domain with no (*unsupervised*) or little labeled data and a larger amount of unlabeled data (*semi-supervised*). For a detailed overview about deep domain adaptation research in medical images, we refer to the very recent survey by Guan et al.^37^. Unsupervised DA gained growing attention in recent years with the advance of generative adversarial networks (GANs)^38^. Adversarial DA applies one or multiple discriminator networks to align the distributions of either the input space at image level, e.g., with CycleGANs^39–41^, the feature space^9,42^, or the output space (segmentation)^43,44^. Furthermore, combinations of these concepts have been proposed, e.g. by Chen et al.^45^. Techniques, that have been originally introduced for semi-supervised learning (SSL), have also been investigated in the context of unsupervised DA. For example, teacher–student models have been used to apply a consistency loss on unlabeled data for spinal cord gray matter segmentation on MRI^46^ and vessel segmentation on retinal fundus images^47^. Another approach combines self-learning with adversarial learning that minimizes discrepancies between feature spaces of $X_S$ and $X_T$ for optical coherence tomography layer segmentation^48^. The segmentation loss and the self-learning curriculum are furthermore guided by uncertainty estimation with a conditional variational auto-encoder. Similarly, methods exist that exploit labeled source and limited labeled target data ($X_T, Y_T$), as well as unlabeled target data for semi-supervised DA with a combination of teacher–student models, CycleGANs and uncertainty guidance induced by Monte Carlo dropout^49^.

A common challenge for medical image segmentation is that the source data—either $X_S$ or $Y_S$—are not always available due to regulations and/or institutional policies on protected health information (PHI), despite all the DA techniques described above require them. Only few works exist, that target this limitation and do not require any images or labels from the source domain. Karani et al.^50^ proposed semi-supervised DA by fine-tuning only batch normalization layers for the adaptation to a new domain. Their method requires, however, that data from multiple source domains are available for training the source model. Bateson et al. propose unsupervised DA for segmentation through entropy-minimization and prior knowledge regularization^51^. A recent study by Xia et al. applied multi-view co-training to multi-organ segmentations in CT datasets^52^. Furthermore, an older but well-established (supervised) DA strategy that relaxes the need for source data, is transfer learning, also known as fine-tuning^53^. For brain lesion segmentation in MRI, the amount of labeled target data could be considerably reduced when the model was initialized with the weights from the source domain and only a limited number of layers was trained on target data^54^. The same effect has been utilized for multiple sclerosis lesion segmentation^55^ and pathological structure segmentation^56^.

While transfer learning is easy to apply and proven effective, a gap between the actual and desired performance remains, especially when only a few labeled target samples are available. To further optimize the performance of transfer learning, we propose to combine SSL with uncertainty-guided self-learning to exploit the information the additional unlabeled images offer. This is inspired by^57^, who found that self-learning is the preferred choice of SSL for transfer learning for classification tasks. However, to the best of our knowledge, no such strategy has been used to address a segmentation or a DA task.

### Contributions

We propose a semi-supervised DA pipeline and applied the method to segment the prostate and critical structures to aid surgical planning. Our main contributions are:

- We investigate the automatic segmentation of the prostate, the EUS and NVB for radical prostatectomy on preoperative MRI. To the best of our knowledge, the EUS and NVB have not been segmented automatically yet.
- We address the problem of domain shift for this task by proposing a semi-supervised DA pipeline. This allows us to perform robust segmentation of the prostate and the critical structures on MRIs acquired outside the institution in which source training data were acquired.
- The proposed pipeline is simple yet effective, does not require the source images and labels, and can be easily adapted to other problems and data. We demonstrate its generic application in additional experiments on pancreas segmentation in CT scans.

## Methods

We split our methods section into two parts. First, we describes our supervised training strategy in the source domain. Second, we outline the proposed semi-supervised domain adaptation method.

### Supervised learning

The supervised leaning uses a labeled dataset $D_l = \{x_i, y_i\}_{i=1}^{n}$. For each image $x_i$ from $X \in \mathbb {R}^{H \times W \times D}$, there exists a ground truth segmentation map $y_i$ from $Y \in \{0,1\}^{H \times W \times D \times C}$, where *W*, *H*, *D* are the dimensions of the volume and *C* defines the number of class labels. In our case, $C=4$ due to the classes prostate, EUS, NVB and background. The network $f(\cdot )$ proposed in this section makes a prediction $p_i$ for an input sample $x_i$, given the learned parameters $\theta$ in training, such that

$$\begin{aligned} p_i = f(x_i, \theta ) \end{aligned}$$

with $p_i \in [0,1]^{H \times W \times D \times C}$. Due to the strong anisotropy of the MR scans (high slice thickness), our supervised method uses an adapted 3D U-Net^32^, which deploys anisotropic MaxPooling in the encoder and anisotropic upsampling in the decoder (see “Appendix 1” for details). We use a network with 16 filters in the first layer and 128 in the bottom-most layer. The last layer of the network uses the softmax activation function and produces a four-channel output for prostate, EUS, NVB and background.

#### Deep ensembles

Network ensembles have been shown to create more robust results than single networks^58,59^. They leverage different minima that CNNs can obtain because networks are subject to randomness during training. In our setting, we employ random parameter initialization, random mini-batches generation during training and different random training/validation splits to increase the local minima variability. We use an ensemble of *k* models and obtain a mean prediction $P_E$ of them.

#### Post-processing

In the first post-processing step, the prediction of the network is thresholded to create a binary prediction. The output is further post-processed with connected components analysis for the EUS and the prostate to ensure topological correctness. The connected component analysis is not applied to the NVB because NVB voxels are not always adjacent in neighboring slices due to the high slice thickness. A connected component analysis would, therefore, risk discarding actual NVB segments.

#### Network training

We trained our network with the negative Dice Similarity Coefficient (DSC) loss function for multi-class segmentations (see “Appendix 2” for details). The Adam optimizer^60^ with a learning rate of $1e^{-03}$ was employed. The network was trained for a maximum of 300 epochs with learning rate decay and with a batch size of 2 on an NVIDIA TitanX GPU. Early stopping was applied if the validation loss did not decrease for 40 epochs. The total number of trainable parameters for the proposed model was 3,197,028.

### Domain adaptation

**Figure 2:**
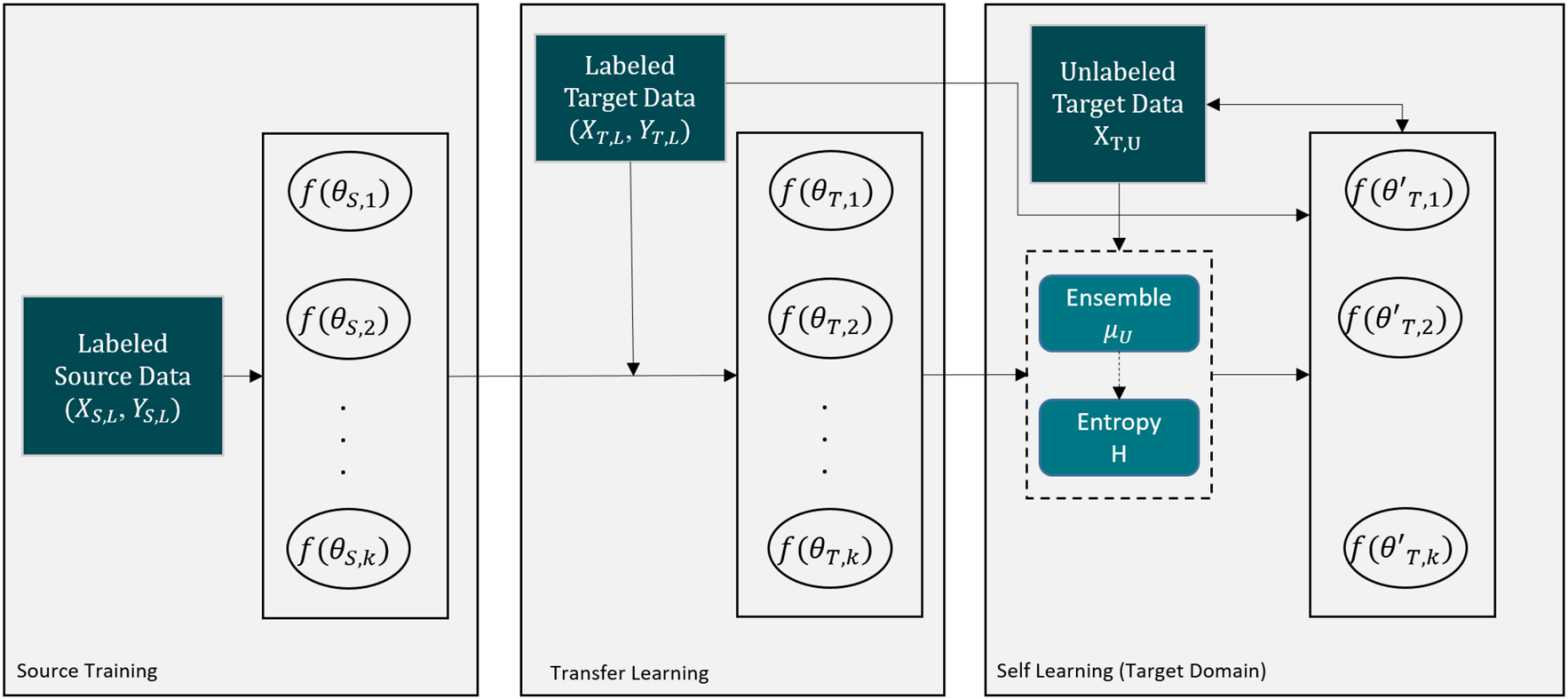
Proposed pipeline for the DA. The ensemble of *k* models is trained in the source domain with the labeled source data. Subsequently, these models are domain adapted by transfer learning with the little labeled data from the target domain and furthermore refined with the self learning routine that includes ensemble-based pseudo labels and entropy guidance.

Our goal is a source-relaxed DA technique composed of two learning concepts: (I) transfer learning as the first stage of DA, and (II) self-learning as a second stage to obtain more information about the distribution of the target domain. To reduce the confirmation bias of self-learning, we propose to use deep ensembles for better segmentation candidates and uncertainty-guidance. In the following, we will describe our proposed method in detail. A summary of the concept of our proposed semi-supervised DA pipeline is depicted in Fig. 2.

For our DA, we have only the source model $f(\theta _S)$ and our target dataset $D_T$ available. As we apply semi-supervised DA, our target dataset consists of *n* labeled volumes $D_{T,L} = \{x_i, y_i\}_{i=1}^n$ and *m* unlabeled volumes $D_{T,U} = \{x_i\}_{i=n+1}^m$.

#### Stage I: Transfer learning

**Figure 3:**
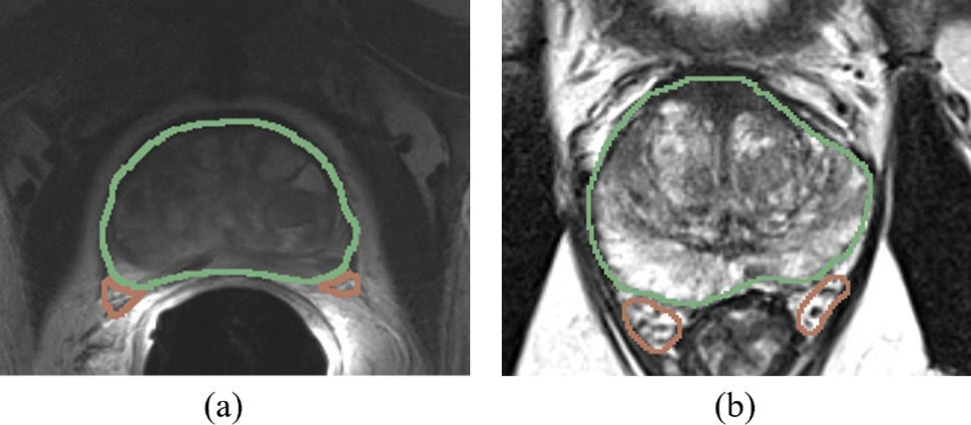
Example images of (**a**) intraoperative endorectal coil acquisition and (**b**) diagnostic pelvic coil acquisition. segmentation of the prostate (green), NVB (brown) and EUS (yellow) are overlayed. It can be seen that the shape, appearance and location of NVB varies as the endorectal coil compresses the tissue during acquisition.

In our scenario, we find large differences in the shape and appearance of the structures between the source and target datasets due to using an endorectal coil in the source dataset. The shape, location, and appearance of the structures-to-segment, particularly the NVB, are changed substantially because of the pressure from the endorectal coil in the source dataset (Fig. 3a, b). For this reason, we propose to have a small amount of labeled pairs ($n \le 10$) in the target domain available.

With the labeled pairs of $(X_L, Y_L) \in D_{T,L}$, we fine-tune our source model $f(\theta _S)$ to a model adapted to the target domain $f(\theta _T)$. As we only have a minimal amount of labeled images, we fix the weights of the decoder and only fine-tune the encoder and the bottom layer weights of the source model. In preliminary experiments on the validation set, this has been working best for a small training dataset.

We fine-tune the models with a reduced learning rate (compared to the fully supervised method) of $1e^{-04}$ until convergence. We apply early stopping if the validation loss did not decrease for 30 epochs. The model weights which give the best validation performance during training is used for the subsequent self-learning.

#### Stage II: Uncertainty-guided self-learning

The transfer learning can be considered as a warm-up phase for the self-learning routine. The fine-tuned model $f(\theta _T)$ is used to make predictions for the unlabeled data $X_{U} \in D_{T,L}$. We post-process these predictions (thresholding and connected components analysis) to improve the segmentation quality. The obtained binary pseudo labels ${Y_U}$ are then fed together with the *n* labeled images as initial pseudo labels into the self-learning stage. Self-learning consists of the cycle of label propagation and retraining the model weights ($f(\theta _T')$) with the newly generated pseudo labels until the performance on validation data does not improve any further. Typically, three to five iterations have to be carried out. In contrast to transfer learning, in the self-learning training procedure, all weights are trained.

For specific voxels of the unlabeled images, no label is given because either none of the classes is above the threshold applied during post-processing or the label has been removed through connected components analysis. Hence, we modify the loss function to account only for voxels that have any label given. We propose to use a partial Dice loss defined as:

$$\begin{aligned} pLoss = - \frac{1}{|C|} \sum _{c \in {C}}{\frac{w_i 2\sum _{i}^{N}P_{c,i}Y_{c,i}M_i}{\sum _{i}^{N}P_{c,i}M_i+\sum _{i}^{N}Y_{c,i}M_i}} \end{aligned}$$

where $Y = Y_U \cup Y_L$ and $M_i$ being defined as:

$$\begin{aligned} M = \left\{ \begin{array}{ll} 0, &{} \sum _C{Y_{c,i}} = 0 \\ 1, &{} \sum _C{Y_{c,i}} > 0 \end{array}\right. \end{aligned}$$

The parameter $w_i$ is a coefficient that weighs the influence of samples on the training. The higher $w_i$, the higher the influence of the samples is. Too high values of $w_i$ for pseudo label samples can lead to a confirmation bias, when too many pseudo label voxels are misclassified, resulting in declined performance on unseen test data. Too small $w_i$ for pseudo label samples may overemphasize the influence of the real ground truth samples, resulting in too little information from the unlabeled data for the gradient update. In this case, the model potentially overfits on the small amount of ground truth labels. In our experiments on the validation set, we found $w=0.5$ for pseudo labels and $w=1.0$ for ground truth labels to be the best setting.

#### Deep ensembles

We propose to use *k* models for better pseudo label generation. We take the mean of the ensemble predictions $\mu _U$ for final prediction outcomes:

$$\begin{aligned} \mu _{U}= \frac{1}{k} \sum _{i=1}^{k}{ f(x, \theta _k)} \end{aligned}$$

Please note that $\theta$ can be either $\theta _T$ (for initial pseudo labels at the beginning of the self-learning stage), or $\theta _T'$ (for the pseudo labels during the self-learning cycle). The mean $\mu _U$ is post-processed to obtain the binary pseudo labels $Y_U$.

#### Uncertainty weighting

Deep ensembling is not only used to improve segmentation accuracy but can also be considered as a mean to estimate the uncertainty measure for the segmentation maps^61^. Hence, we utilize the entropy of ensemble predictions for a sample-wise uncertainty weighting for our loss function to reduce the impact of low quality pseudo labels. The entropy is computed as:

$$\begin{aligned} H_i = -\sum _{i=1}^{c} \mu _c \log {\mu _c} \end{aligned}$$

The case-based entropy is then normalized as:

$$\begin{aligned} H_i = \frac{H_i}{\max _i{H_i}} \end{aligned}$$

For our method with uncertainty-weighting, the sample weights for labeled data are $w_i=1$ and the weight for the pseudo-label samples is set to $w_{i}=1-H_i$. We used an ensemble of $k=5$ models for the uncertainty generation.

### Data

For the evaluation of our method, we use multiple datasets that we will describe in the following. To evaluate our method for the critical structure segmentation for prostate cancer therapy, we used two datasets, that represent the source and target dataset for this task. For investigating the generalization capability of our DA framework, we used different abdominal CT datasets as source and target data.

#### Prostate MRI

##### Source data:

Sixty-two patients who were scheduled for robot-assisted laparoscopic prostatectomy (RALP) underwent preoperative multiparametric MRI in a 3-T MR imager (Signa HDxt 3.0 T; GE Healthcare, Milwaukee, Wis) with both endorectal (Medrad, Warrendale, Pa) and pelvic phased-array coils. As part of the protocol, an axial multi-slice T2-weighted image was acquired using a 2D turbo spin sequence with an in-plane spacing of $0.27 \times 0.27$ mm and a slice thickness of 3 mm. The retrospective analysis of preoperative multiparametric MRI data for this study was approved by the institutional review board of the hospital (Brigham and Women’s Hospital, Boston, MA, USA) and is in accordance with relevant guidelines and regulations (Health Insurance Portability and Accountability Act). All the subjects were given written informed consent prior to enrollment. In the following, we will refer to this dataset as the BWH dataset. The gland, NVB, and EUS were manually segmented by Reader 1, an expert radiologist (C.M.T.), using the Editor tool on 3D Slicer^62^. For evaluating the inter-reader variability and the performance of the automatic segmentation, a second label dataset was created by Reader 2, a research fellow with a medical background and two years of experience in reading prostate MRI (A.Z.). For training, only the manual labels of Reader 1 were used as target labels $Y_S$.

##### Target data:

For DA, we used the Prostate-3T dataset^63^. The dataset consists of 64 axial T2w scans that were acquired on a 3T Siemens TrioTim using only a pelvic phased-array coil. The slice thickness varied between 3.0 and 5 mm and the spacing was between 0.5 and 0.625 mm. We selected 25 scans from this dataset for which either segmentations of NVB or peripheral and transition zone of the prostate are available through the NCI-ISBI 2013 challenge^64^ and the Cancer Imaging Archive^65^. The prostate segmentation for the NCI-ISBI 2013 challenge is defined as the union of transition and peripheral zone segmentations. A medical student (B.L.) outlined the structures that were not provided by any of these two ground truth sources. In the end, for each of these 25 volumes, a three-class segmentation existed.

##### Training, validation and test split:

The data of the source domain was split into training (n = 46) and test data (n = 16). The test data has been held out from the experiments until the final evaluation of the methods. The source training data was split in a five-fold-cross-validation manner resulting in about 36 training images and 10 validation images. The performance of the method for each fold was computed on the 16 hold-out test cases of the BWH dataset.

For the target dataset, the scans were split into labeled training ($n=15$) and held-out test data ($n=10$). We carried out three-fold cross-validation on the training data and evaluate the performance of the method for each fold on the test cases to obtain robust estimate of the method’s performance for different training data distributions. We use $n=5$ and $n=10$ labeled training and five validation images (as determined by the fold split) as well as the remaining unlabeled images of the dataset for our semi-supervised DA. We empirically set the lowest number of labeled training samples to $n=5$, because the method should see some variance in the provided labeled dataset (e.g., organ size, relationship of the organ-to-segment and surrounding organs, diseases, imaging contrasts, noise, bias fields etc.). However, it should be possible to run the method with even less number of labeled training samples, but presumably the results’ quality will decrease in this scenario.

##### Pre-processing and augmentation:

All volumes were resampled to a spacing of $0.5\,\times 0.5\,\times \,3.0$ mm. A bounding box ROI of the prostate was extracted from the center of the volume by cropping the volume to a size of $184\,\times \,184\,\times \,32$. Prior to normalization of image intensity to an interval of [0,1], the intensities were cropped to the first and 99th percentile. The training data was augmented by left-right flipping of the volume.

#### Pancreas CT

##### Source data:

For the source domain, we used two abdominal datasets: the TCIA Pancreas-CT dataset^65–67^ and the Beyond the Cranial Vault Abdomen dataset^68,69^ (BTCV). In the TCIA dataset, contrast enhanced 3D CT scans at the National Institutes of Health Clinical Center (Bethesda, MD, USA) from pre-nephrectomy healthy kidney donors were acquired. The BTCV dataset was acquired during portal venous contrast phase at the Vanderbilt University Medical Center (Nashville, TN, USA) from metastatic liver patients or post-operative ventral hernia patients. We used the publicly available segmentations^70^ for the TCIA dataset (n = 47) and the BTCV abdomen dataset (n = 42) as our source training data.

##### Target data:

The dataset for the target domain was derived from the Medical Segmentation Decathlon Challenge^71^ (MSD). The dataset consists of portal venous phase CT scans that were acquired from patients undergoing resection of pancreatic masses at Memorial Sloan Kettering Cancer Center (New York, USA). 281 labeled cases are publicly available in this challenge dataset. For the pancreas segmentation, the domain shift is not only limited to differences in image appearance, but additionally covers the different distributions of healthy pancreas (source domain) and cancerous pancreas (target domain).

##### Training, validation and test split:

We split the source domain into 14 test cases and 75 training cases. For the latter we perform 5-fold cross validation (60/15 training/validation split) to obtain five models in the source domain. For the target dataset we set the same 81 cases as hold-out test cases as in^52^, the remaining 200 cases were used as training cases for the target domain. For the fully supervised target domain model we used a training/ validation split of 80%/20%, which results in 160 training and 40 validation cases. For our semi-supervised domain adaptation, we randomly selected $n=10$ and $n=5$ labeled scans for training and 10 labeled scans for validation from the respective set. The remaining training images were used as unlabeled training input. We repeat the random selection of subsets three times, to reduce the bias that small subsets can have on the model performance.

##### Pre-processing and augmentation:

The scans are resampled to a common spacing of $1.0 \times 1.0 \times 3.0$ mm and cropped to a ROI of [200, 128, 48] surrounding the GT pancreas segmentation. The intensities are first clipped to a range of $[-300,300]$ and subsequently normalized to zero mean and unit variance. We applied random geometric (translate, scale) and intensity (Gaussian noise, Gaussian blurring) transformations as online augmentations.

### Evaluation measures

We evaluated our approaches with the DSC and the average boundary distances (ABD) between surface points of both volumes. DSC is defined as:

$$\begin{aligned} DSC = \frac{2|X \cap Y|}{|X|+|Y|}, \end{aligned}$$

with *X* being the predicted and *Y* being the ground truth voxels. The ABD is defined as:

$$\begin{aligned} ABD(X_{S}, Y_{S}) = \frac{1}{|X_S|+|Y_S|} \left( \sum _{x \in {X_S}} \min _{y \in Y_S}{} \textit{dist}(x,y) \right. \left. + \sum _{y \in {Y_S}} \min _{x \in X_S}{} \textit{dist}(y,x) \right) , \end{aligned}$$

where $X_S$ and $Y_S$ are the sets of surface points of the predicted and the ground truth segmentation and *dist* is the Euclidean distance operator.

## Results

### Supervised learning

The results for the automatic segmentation of prostate, EUS and NVB are compared against Reader 1 in Table 1. We evaluated the average performance of the folds for a single network (sCNN), the performance of the ensembling of models (eCNN) as well as the manual performance of a second reader in comparison to the first reader who created the ground truth segmentations.

The average performance of sCNN across the folds are DSCs of 0.877, 0.648 and 0.558 for prostate, EUS, and NVB. The ensemble eCNN improved the results to DSCs of 0.893, 0.683, and 0.583. Both approaches obtain better results compared to the inter-reader evaluation, which only achieved DSCs of 0.863, 0.465, and 0.546 for the prostate, EUS and NVB, respectively. Although the DSC values for EUS and NVB may appear quite low, the results’ quality is better than expected from these values. As overlap-based metrics generally have lower values for smaller structures, we refer to the ABD values for interpretation. The ABD for the NVB was 1.27 mm and 1.36 mm for the EUS for eCNN. Visual inspection supported these findings.

To quantify the effect of the domain shift of our source model’s performance in the target domain, we apply the single network (sCNN) to the Prostate-3T data. Average results for this experiment can be found in Table 2. The DSC for the prostate decreases from 0.877 on BWH data to 0.638 on Prostate-3T data. Similarly, the DSC for EUS decreases from 0.648 to 0.291 and the DSC for NVB drops from 0.558 to 0.177.

**Table 1:** Comparison of segmentation results on BWH test data of the automatic single (sCNN) and ensemble CNN (eCNN) prediction against manual segmentation by Reader 1.

	Prostate		EUS		NVB	
	Dice	ABD	Dice	ABD	Dice	ABD
Reader 1 versus sCNN	0.877	1.17	0.648	1.54	0.558	1.46
Reader 1 versus eCNN	0.893	0.98	0.683	1.36	0.583	1.27
Reader 1 vs 2	0.863	1.61	0.465	2.10	0.546	1.68

Manual segmentation by Reader 2 is also compared against Reader 1. ABD is given in millimeter (mm).

### Domain adaptation

**Figure 4:**
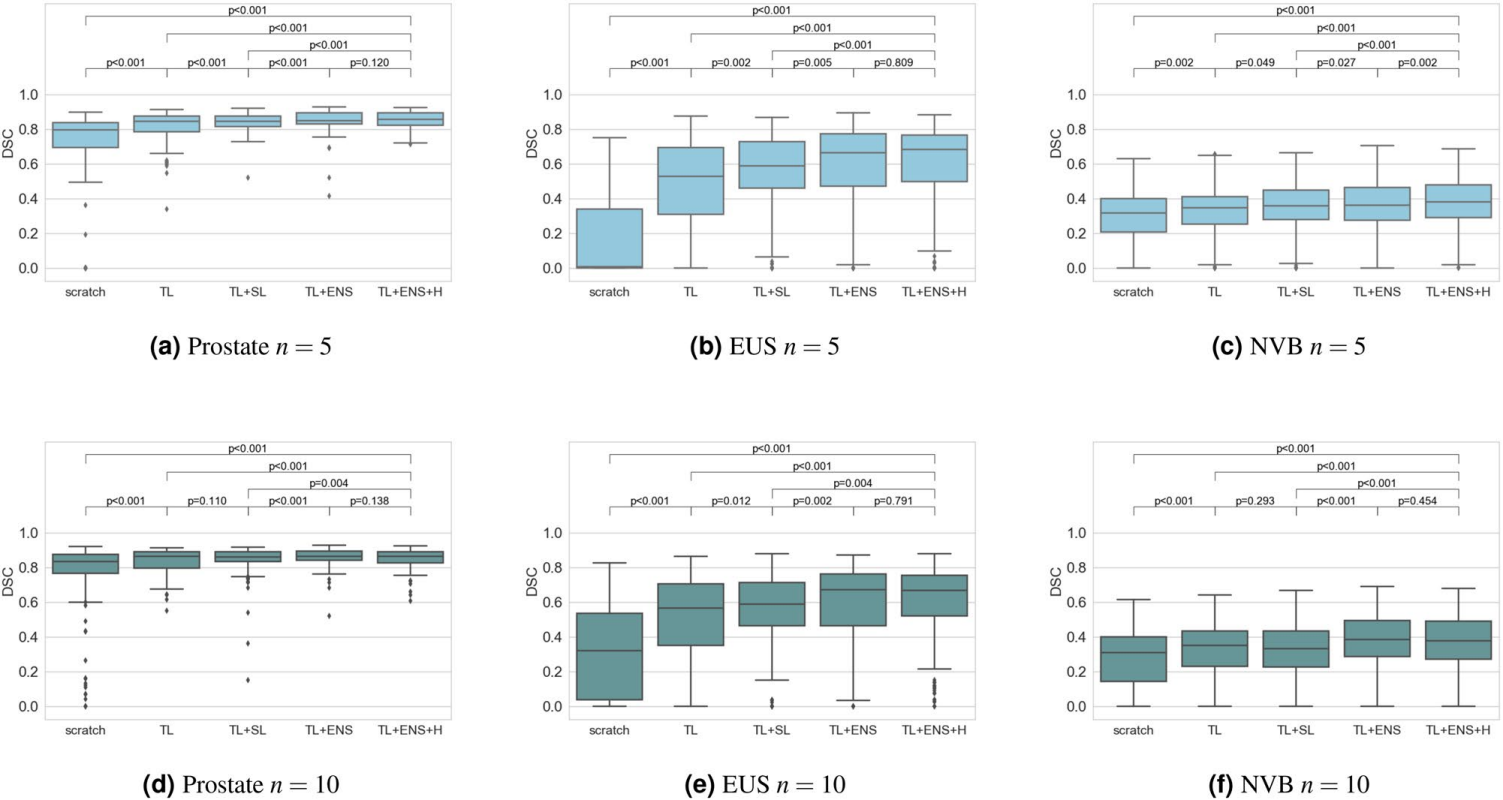
Boxplots for evaluation of the methods with $n=5$ and $n=10$ labeled images in target domain. P-values for the statistical significant differences between the methods are provided in the top of the plots. Due to the small test sample size, we utilized the results of the five models for the the 3-fold cross validation. This way, we obtain $5*3=15$ individual results for each sample case and each method, allowing for statistical evaluation despite the small test set size.

**Figure 5:**
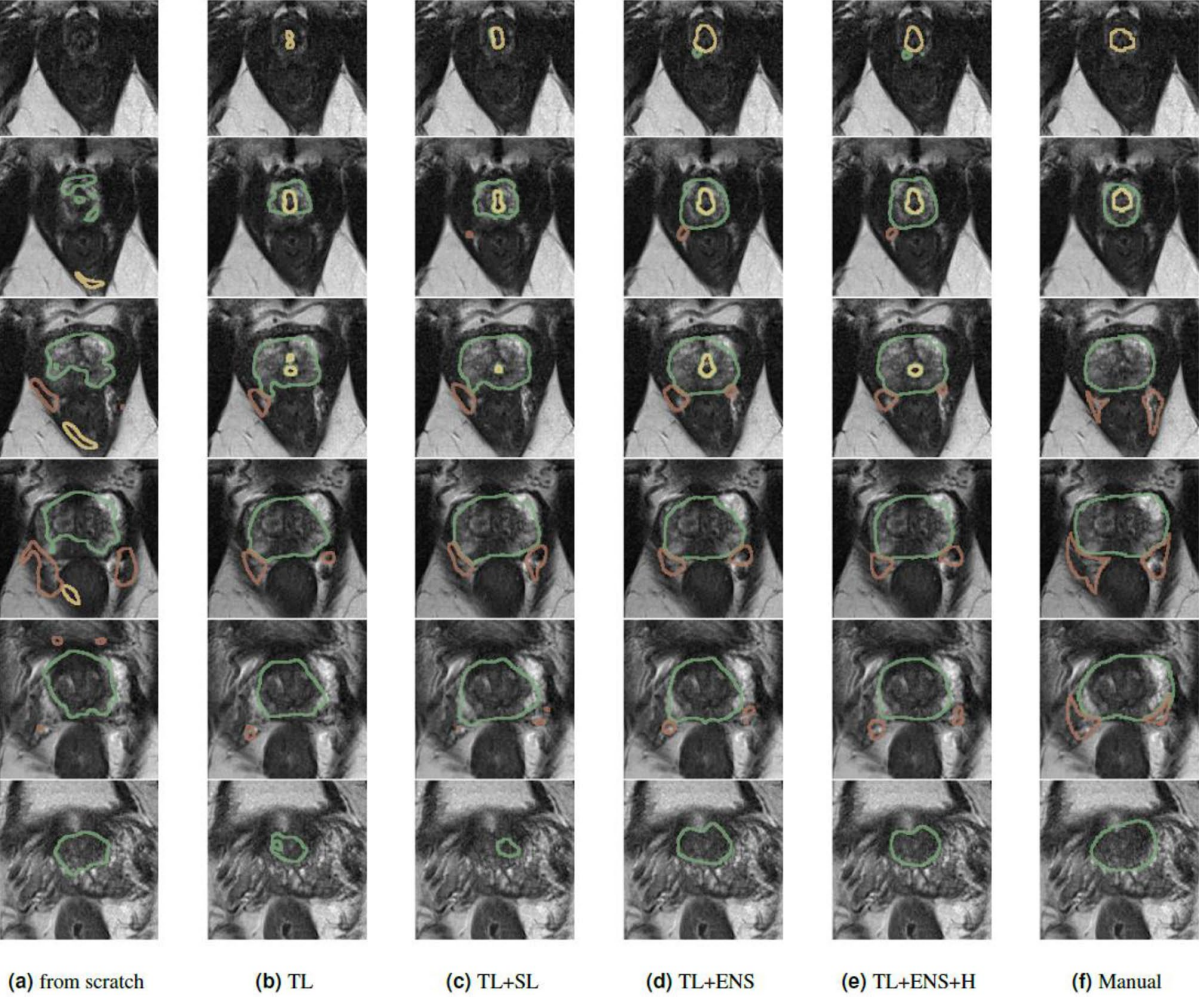
Example segmentation result of one case for the discussed approaches. The quality of segmentation improves over the added features of our method. The DC for TL $+$ ENS $+$ H approach is 0.817 and 0.726 for training from scratch. For the EUS the DC is 0.706 and 0.0, respectively. NVB obtains a DC of 0.392 for training from scratch and a DC of 0.488 for the proposed TL $+$ ENS $+$ H approach.The training for the CNNs applied to this case was run with $n=5$ images.

We assess the segmentation quality for training from scratch, transfer learning and our semi-supervised DA technique. Additionally, we performed an ablation study to evaluate the impact of the ensembling of models (ENS) and the uncertainty-weighting (H). Example outcomes are shown in Fig. 5. The quantitative results are summarized in Table 2 with box-plots of their DSC’s distribution in Fig. 4 with correpsonding *p* values (Wilcoxon signed-rank test).

**Table 2:** Evaluation results for the source model, training from scratch and the proposed DA method with its ablation study on Prostate-3T test data.

Method	Labeled data	Prostate		EUS		NVB	
		DSC	ABD	DSC	ABD	DSC	ABD
From scratch	$n=5$	0.694	4.44	0.177	10.39	0.303	7.98
	$n=10$	0.760	2.55	0.320	3.51	0.280	6.25
TL	$n=5$	0.814	1.98	0.480	2.88	0.337	4.98
	$n=10$	0.834	1.61	0.495	2.00	0.335	4.11
TL $+$ SL	$n=5$	0.843	1.57	0.546	1.73	0.350	4.21
	$n=10$	0.841	1.53	0.552	1.55	0.382	3.39
TL $+$ ENS	$n=5$	0.849	1.51	0.578	1.43	0.363	3.83
	$n=10$	0.860	1.36	0.596	1.33	0.382	3.39
ENS $+$ H	$n=5$	0.831	1.86	0.535	1.87	0.355	4.46
	$n=10$	0.850	1.54	**0.598**	1.51	0.379	3.61
Ours (TL $+$ ENS $+$ H)	$n=5$	0.855	1.45	0.580	1.62	0.378	3.37
	$n=10$	0.855	1.42	0.593	1.40	0.374	3.63
Ours (Majority)	$n=5$	0.865	1.33	0.592	**1.21**	**0.387**	3.48
	$n=10$	**0.866**	**1.29**	0.591	1.41	0.381	**3.24**

Results are given as Dice Coefficient (DSC) and average boundary distance (ABD in mm). Majority (TL $+$ ENS $+$ H) denotes the approach, where the ensemble of models from our TL $+$ ENS $+$ H is used to generate a majority vote as outcome. Best results are marked bold.

For $n=5$, we found that the mean DSC increased with each step of our proposed pipeline for the data of our target domain. For the prostate, the mean DSC was 0.694 after training from scratch on the five labeled images. It increased to 0.814, 0.843, 0.849, and to 0.855 with transfer learning (TL), the additional self-learning (TL + SL), the ensemble-based self-learning without uncertainty (TL + ENS), and with uncertainty (TL + ENS + H), respectively. When applying majority voting of the ensemble that resulted from TL + ENS + H, the results could further be improved to a DSC of 0.865 for the prostate. Similar to the prostate, we could also observe improvements for NVB and EUS with each step of our domain adaptation pipeline.

Also for $n=10$, improvements through the self-learning (SL) and ensembling (ENS) components are noted in the results. For this setting, though, the incorporation of entropy (H) as uncertainty guidance did not contribute to any improvement. We assume, that the model predictions together with their post-processing are already good enough for the self-learning.

Similar to transfer learning, variants of uncertainty-guided self-learning has been proposed as state-of-the-art method, e.g. by Wang et al.^48^ for (unsupervised) domain adaptation. Because the works described in the literature need the data from the source domain to be available, we evaluated our variant of uncertainty-guided self-learning (ENS + H) without the TL component, to compare against another state-of-the-art method. As can be seen in Table 2, this technique works substantially better than pure TL, but our method that combines both techniques, works considerably better in particular for $n=5$ labeled training cases.

The results for the NVB are low in general for all methods proposed in the DA section. This is likely because the NVB is a thin, tubular structure and is often obscured by the surrounding structures and image artifacts resulting in inconsistent labeling between the readers. Furthermore, the analysis of the connected components is not applicable such that some predictions far off the right location do not get filtered out for pseudo labels. The other two structures are in the range of inter-reader variability if we compare to the results from the two observers in the source domain.

#### Generalization capability

To investigate the generalization capability, we investigated our DA method for pancreas segmentation in CT scans. The results are summarized in Table 3. We see that a considerable domain shift exists as the source model’s performance drops from a DSC of 0.694 (source test data) to a DSC of 0.638 on the target test data. The performance could be improved to a DSC of 0.726 with only five labeled target cases as (labeled) training data. This corresponds to a relative improvement of 13.8%. Applying the ensembling strategy to our method, the average performance on the test data can be improved to 0.732 for the $n=5$ setting. If we increase our labeled training set size to $n=10$, we can observe an improvement in the transfer learning results. However, the complete DA pipeline does not lead to much better results than for the $n=5$ setting. This indicates the high potential that the analysis of the unlabeled data in the target domain can have.

Because we used the same test dataset as the work by Xia et al.^52^, we can make a relative comparison for the performance gain to this state-of-the-art technique. The source model from Xia et al. had a performance DSC of 0.817 in the source domain which decreased to 0.702 in the target domain. Through their multi-view co-training DA method, they could achieve a DSC of 0.749 with access to the labeled data in the source domain and a DSC of 0.744 in the source-relaxed DA setting. Thus, for the source-relaxed setting, they achieved a relative performance gain of 5.9%. Although there exist some differences in the implementation of their method which make a direct comparison impossible (other backbone architecture, additional segmentation of other organs in the source domain), these results (relative performance gain of 5.9% vs. 13.8%) indicate the effectiveness of our method and motivate using few labeled samples of the target domain.

**Table 3:** Results (DSC) for the pancreas CT datasets.

	From scratch	Transfer learning	Ours	Ours (Majority)	Source model (source)	Source model (target)	Target model (target)
$\hbox {n}=5$	0.449	0.678	0.726	0.732	0.694	0.638	0.773
$\hbox {n}=10$	0.524	0.690	0.729	0.733			

## Discussion

Our study demonstrated the feasibility of automatic CNN-based segmentation of the prostate, NVB, and EUS, relevant to treatment planning. We showed that the anisotropic variant of the 3D U-Net performs as well as an experienced human reader in segmenting those structures. To the best of our knowledge, this is the first study to address the automatic segmentation of the NVB and EUS.

The strongly decreased performance of the model on the unseen Prostate-3T dataset highlights the necessity for a technique that adapts the model to the target data distribution. We proposed a simple yet effective DA technique that combines transfer learning and uncertainty-guided self-learning. DA is crucial for the widespread clinical use of 3D-model-based surgical planning, given that the characteristics of prostate MRI heavily depend on the types of the scanner and coils used, MR sequence, and imaging parameters. Without DA, one would need to create a model for each clinical site involving manual labeling of tens of volumes as the training dataset. In contrast, our study has demonstrated that we only need labeled images as few as $n=5$ to transfer learning to the new clinical site, making the routine use of 3D-model-based surgical planning more feasible and practical.

The advantage of our DA method over many others is that it only requires the model that was trained on source data. This is particularly helpful when the entire source dataset cannot be shared with other clinical sites due to the size, or institutional and/or regulatory rules over the protection of PHI. Our DA method is simple to apply and does not require any network modification like adversarial training as in^44^ which induces more computational resources or patch-based approaches that do not capture the volume as a whole. Also, no prior knowledge about organ to segment as in^51^ is needed, making it easy to apply to other tasks.

Although our evaluation showed that domain adapted models performed well in the target domain for most structures, our study has limitations. First, the ensembling of source models, which aims to provide better pseudo label candidates and uncertainty measures, may not be applicable when only one source model is available. In this case, ensembling could alternatively be achieved for example by Monte-Carlo dropout^72^, different subsets of labeled/unlabeled data from the target domain, or different minima during training from only one network^73^. Furthermore, a combination of different training schemes as different regularizations, different loss functions, different learning rates could be employed to generate models with differentiating minima. Second, we used an ensemble size of $k=5$, which is relatively small but a compromise between computation time and performance. If enough computation resource is available, the number of models could be increased and performance may improve further.

The ability to segment substructures of the prostate will have a broader impact on PCa treatment. 3D geometric models of the EUS and NVB based on the proposed segmentation technique will allow detailed treatment planning of PCa, for example with focal therapy. For this application, the segmentation technique would need to be extended to include other surrounding structures, such as the rectal and bladder walls, which must also be protected from accidental damage. However, the proposed method could be easily extended to include the structures around the prostate relevant to the therapy planning.

We observed a rather low performance of our method for the NVB structure in the target domain. While the endorectal coil especially affects the shape and appearance of this structure, the low performance is presumably caused to a large extent by disagreement of the different readers involved for the NVB segmentation. Therefore, future work should include a consensus segmentation of the NVB among multiple readers on publicly available datasets, to have a more consistent ground truth for our DA method evaluation.

## Conclusion

This study demonstrated automatic segmentation of critical structures for PCa treatment, including the prostate, EUS, and NVB based on an anisotropic CNN. Moreover, we proposed a new DA strategy that combines transfer learning and uncertainty-guided self-learning. The proposed strategy allows applying a trained network to another domain, e.g., another scanner or another acquisition protocol, with minimum quality loss, making automatic segmentation suitable for clinical applications, where the sharing of patient data is often highly restricted. Our model achieves performance comparable to an experienced human reader in the source domain, and the DA gains performance similar to human readers for the prostate and the EUS. The high performance of CNNs allows for a more precise planning of PCa therapy and thus has the potential to reduce the complications in PCa interventions. Finally, we demonstrated the generic application of our DA framework by investigating its performance on another challenging task and data, namely pancreas CT segmentation.

### Supplementary information

Supplementary Information.

## Figures and Tables

**Figure 6:**
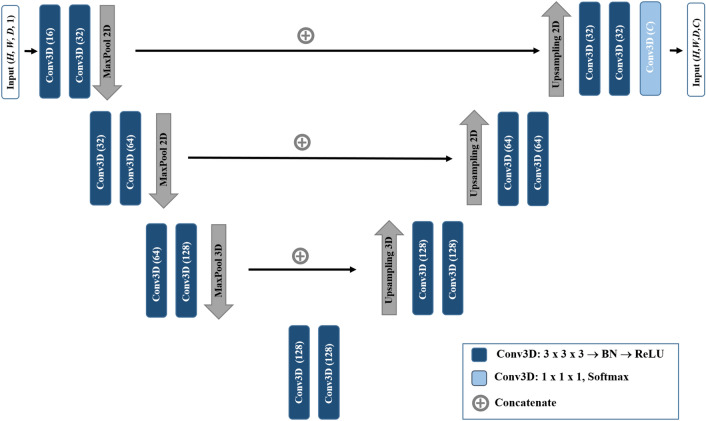
Anisotropic network architecture with settings for the prostate structure segmentation. The numbers in the brackets in the convolutional layers correspond to the number of feature maps returned by the convolutional layer.

## Data Availability

The target dataset is a publicly available challenge dataset (https://wiki.cancerimagingarchive.net/display/Public/Prostate-3T) and the segmentations created for this data during our study are provided as supplemental material. The BWH (source) dataset is not publicly available due to restrictions in the IRB-approved protocol under which the data were obtained. The pancreas datasets are publicly available (see corresponding references). The trained models and our code can be shared upon request.

## Appendix 1: Network architecture

The anisotropic newtork architecture used in our experiments is depicted in Fig. 6.

## Appendix 2: DSC loss function

The DSC loss function for varying number of classes is:

$$\begin{aligned} loss = - \frac{1}{|C|} \sum _{c \in {C}}{\frac{2\sum _{i}^{N}P_{c,i}G_{c,i}+\epsilon }{\sum _{i}^{N}P_{c,i}+\sum _{i}^{N}G_{c,i}+\epsilon }}, \end{aligned}$$

with $N$ being the total number of voxels and $p_{c,i}$ the predicted voxels and $g_{c,i}$ the ground truth binary voxels of class *c*. $\epsilon$ is a small constant that guarantees numerical stability.

## References

[1] Siegel RL, Miller KD, Jemal A (2020). Cancer statistics, 2020. CA Cancer J. Clin..

[2] Cooperberg MR, Lubeck DP, Meng MV, Mehta SS, Carroll PR (2004). The changing face of low-risk prostate cancer: Trends in clinical presentation and primary management. J. Clin. Oncol..

[3] Hu JC (2009). Comparative effectiveness of minimally invasive vs open radical prostatectomy. JAMA.

[4] Nguyen LN (2017). The risks and benefits of cavernous neurovascular bundle sparing during radical prostatectomy: A systematic review and Meta-Analysis. J. Urol..

[5] Mungovan SF (2017). Preoperative membranous urethral length measurement and continence recovery following radical prostatectomy: A systematic review and meta-analysis. Eur. Urol..

[6] Kozikowski M, Malewski W, Michalak W, Dobruch J (2019). Clinical utility of MRI in the decision-making process before radical prostatectomy: Systematic review and meta-analysis. PLoS ONE.

[7] Wang, S. *et al.* The use of three-dimensional visualization techniques for prostate procedures: A systematic review. *Eur. Urol. Focus* (2020). 10.1016/j.euf.2020.08.002 32873515

[8] Dai Z (2020). Segmentation of the prostatic gland and the intraprostatic lesions on multiparametic magnetic resonance imaging using mask region-based convolutional neural networks. Adv. Radiat. Oncol..

[9] Dou, Q., Ouyang, C., Chen, C., Chen, H. & Heng, P.-A. Unsupervised cross-modality domain adaptation of convents for biomedical image segmentations with adversarial loss. In *Proc. Int. Joint Conf. Artif. Intell. (IJCAI)*, 691–697 (AAAI Press, 2018).

[10] Mehrtash, A. *et al.* Deepinfer: Open-source deep learning deployment toolkit for image-guided therapy. In *Proc. SPIE Int. Soc. Opt. Eng.*, vol. 10135 (2017). 10.1117/12.2256011 PMC5467894 28615794

[11] Hosny, A. *et al.* Modelhub.ai: Dissemination platform for deep learning models. *CoRR* (2019).

[12] Rieke N (2020). The future of digital health with federated learning. NPJ Digit. Med..

[13] Ghose S (2012). A survey of prostate segmentation methodologies in ultrasound, magnetic resonance and computed tomography images. Comput. Methods Progr. Biomed..

[14] Ronneberger, O., Fischer, P. & Brox, T. U-net: convolutional networks for biomedical image segmentation. In *Med. Image Comput. Comput. Assist. Interv.*, 234–241 (2015).

[15] Çiçek, Ö., Abdulkadir, A., Lienkamp, S. S., Brox, T. & Ronneberger, O. 3D U-Net: learning dense volumetric segmentation from sparse annotation. In *Med. Image Comput. Comput. Assist. Interv.*, 424–432 (2016).

[16] Zhu, Q., Du, B., Turkbey, B., Choyke, P. L. & Yan, P. Deeply-supervised CNN for prostate segmentation. In *IEEE Proc. Int. Jt. Conf. Neural Netw. (IJCNN)*, 178–184 (2017).

[17] Wang B (2019). Deeply supervised 3D fully convolutional networks with group dilated convolution for automatic MRI prostate segmentation. Med. Phys..

[18] Wang, B. *et al.* Automatic MRI prostate segmentation using 3D deeply supervised FCN with concatenated atrous convolution. *SPIE Med. Imaging Comput. Aided Diagnosis*. 10.1117/12.2512551 (2019).

[19] Cheng, R. *et al.* Deep learning with orthogonal volumetric HED segmentation and 3D surface reconstruction model of prostate MRI. In *IEEE Proc. Int. Symp. on Biomed. Imaging ISBI*, 749–753 (2017).

[20] Meyer, A. *et al.* Anisotropic 3D multi-stream CNN for accurate prostate segmentation from multi-planar MRI. *Computer Methods and Programs in Biomedicine*. 10.1016/j.cmpb.2020.105821 (2020). 10.1016/j.cmpb.2020.105821 33218704

[21] Yu, L., Yang, X., Chen, H., Qin, J. & Heng, P.-A. Volumetric ConvNets with mixed residual connections for automated prostate segmentation from 3D MR images. *AAAI Conf. Artif. Intell.* 66–72 (2017).

[22] Hossain MS, Paplinski AP, Betts JM (2018). Residual semantic segmentation of the prostate from magnetic resonance images. Int. Conf. Neural Inf. Proc..

[23] Jia H (2020). 3D APA-Net: 3D adversarial pyramid anisotropic convolutional network for prostate segmentation in MR images. IEEE Trans. Med. Imaging.

[24] Hassanzadeh T, Hamey LGC, Ho-Shon K (2019). Convolutional neural networks for prostate magnetic resonance image segmentation. IEEE Access.

[25] Yuan, Y. *et al.* Prostate segmentation with encoder–decoder densely connected convolutional network (Ed-Densenet). In *IEEE Int. Symp. Biom. Imaging (ISBI)* 434–437. 10.1109/ISBI.2019.8759498 (2019).

[26] Zhu Q, Du B, Yan P (2020). Boundary-weighted domain adaptive neural network for prostate MR image segmentation. IEEE Trans. Med. Imaging.

[27] Zhu, Q., Du, B., Wu, J. & Yan, P. A deep learning health data analysis approach: automatic 3D prostate MR segmentation with densely-connected volumetric ConvNets. In *IEEE Proc. Int. Jt. Conf. Neural Netw. (IJCNN)* 1–6. 10.1109/IJCNN.2018.8489136 (2018).

[28] To MNN, Vu DQ, Turkbey B, Choyke PL, Kwak JT (2018). Deep dense multi-path neural network for prostate segmentation in magnetic resonance imaging. Int. J. Comput. Assist. Radiol. Surg..

[29] Liu, Q., Fu, M., Gong, X. & Jiang, H. Densely Dilated Spatial Pooling Convolutional Network using benign loss functions for imbalanced volumetric prostate segmentation. *CoRR* (2018).

[30] Brosch, T., Peters, J., Groth, A., Stehle, T. & Weese, J. Deep learning-based boundary detection for model-based segmentation with application to mr prostate segmentation. *Med. Image Comput. Comput. Assist. Interv.* 515–522 10.1007/978-3-030-00937-3_59 (2018).

[31] Yang, X. *et al.* A 3D neurovascular bundles segmentation method based on MR-TRUS deformable registration. In *SPIE Med. Imaging 2015: Image Processing* 941319 10.1117/12.2077828 (2015). 10.1117/12.2077828 PMC6715139 31467458

[32] Meyer, A. *et al.* Towards patient-individual PI-Rads v2 sector map: CNN for automatic segmentation of prostatic zones from T2-weighted MRI. *IEEE Int. Symp. Biomed. Imaging (ISBI)* 696–700 (2019).

[33] Hambarde P (2019). Radiomics for peripheral zone and intra-prostatic urethra segmentation in MR imaging. Biomed. Signal Process. Control.

[34] Tajbakhsh N (2020). Embracing imperfect datasets: a review of deep learning solutions for medical image segmentation. Med. Image Anal..

[35] Zhang L (2020). Generalizing deep learning for medical image segmentation to unseen domains via deep stacked transformation. IEEE Trans. Med. Imaging.

[36] Liu, Q., Dou, Q. & Heng, P.-A. Shape-aware meta-learning for generalizing prostate MRI segmentation to unseen domains. In *Med. Image Comput. Comput. Assist. Interv.* 475–485 (2020).

[37] Guan, H. & Liu, M. Domain adaptation for medical image analysis: a survey. arXiv preprint arXiv:2102.09508 (2021). 10.1109/TBME.2021.3117407 PMC9011180 34606445

[38] Goodfellow, I. *et al.* Generative adversarial nets. *Adv. Neural Inf. Process. Syst.* 2672–2680 (2014).

[39] Zhu, J.-Y., Park, T., Isola, P. & Efros, A. A. Unpaired image-to-image translation using cycle-consistent adversarial networks. In *Proc IEEE Comput. Soc. Conf. Comput. Vis. Pattern Recognit.* 2223–2232 (2017).

[40] Huo, Y. *et al.* Adversarial synthesis learning enables segmentation without target modality ground truth. *IEEE Int. Symp. Biomed. Imaging (ISBI)* 1217–1220 (2018). 10.1109/isbi.2018.8363790 PMC12462902 41018946

[41] Chen, C., Dou, Q., Chen, H. & Heng, P.-A. Semantic-aware generative adversarial nets for unsupervised domain adaptation in chest X-ray segmentation. In *International Workshop on Machine Learning in Medical Imaging* 143–151 (2018).

[42] Kamnitsas K (2017). Unsupervised domain adaptation in brain lesion segmentation with adversarial networks. Inf. Process. Med. Imaging.

[43] Tsai, Y.-H. *et al.* Learning to adapt structured output space for semantic segmentation. In *IEEE Comput. Conf. Comput. Vis. Pattern Recognit.* 7472–7481 (2018).

[44] Yan W, Wang Y, Xia M, Tao Q (2019). Edge-guided output adaptor: highly efficient adaptation module for cross-vendor medical image segmentation. IEEE Signal Process. Lett..

[45] Chen, C., Dou, Q., Chen, H., Qin, J. & Heng, P. A. Unsupervised bidirectional cross-modality adaptation via deeply synergistic image and feature alignment for medical image segmentation (2020). arXiv:2002.02255. 10.1109/TMI.2020.2972701 32054572

[46] Perone CS, Ballester P, Barros RC, Cohen-Adad J (2019). Unsupervised domain adaptation for medical imaging segmentation with self-ensembling. Neuroimage.

[47] Fotedar, G., Tajbakhsh, N., Ananth, S. & Ding, X. Extreme consistency: Overcoming annotation scarcity and domain shifts. In *Med. Image Comput. Comput. Assist. Interv.* 699–709 (2020).

[48] Wang, J. *et al.* Uncertainty-guided domain alignment for layer segmentation in oct images. arXiv preprint arXiv:1908.08242 (2019).

[49] Li, K., Wang, S., Yu, L. & Heng, P.-A. Dual-teacher++: Exploiting intra-domain and inter-domain knowledge with reliable transfer for cardiac segmentation. *IEEE Trans. Med. Imaging* (2020). 10.1109/TMI.2020.3038828 33201808

[50] Karani, N., Chaitanya, K., Baumgartner, C. & Konukoglu, E. A lifelong learning approach to brain MR segmentation across scanners and protocols. In *Med. Image Comput. Comput. Assist. Interv.* 476–484 (2018).

[51] Bateson, M., Kervadec, H., Dolz, J., Lombaert, H. & Ben Ayed, I. Source-relaxed domain adaptation for image segmentation. In *Med. Image Comput. Comput. Assist. Interv.* 490–499 (2020).

[52] Xia Y (2020). Uncertainty-aware multi-view co-training for semi-supervised medical image segmentation and domain adaptation. Med. Image Anal..

[53] Tajbakhsh N (2016). Convolutional neural networks for medical image analysis: Full training or fine tuning?. IEEE Trans. Med. Imaging.

[54] Ghafoorian M (2017). Transfer learning for domain adaptation in MRI: Application in brain lesion segmentation. Med. Image Comput. Comput. Assist. Interv..

[55] Valverde S (2019). One-shot domain adaptation in multiple sclerosis lesion segmentation using convolutional neural networks. Neuroimage Clin..

[56] Kaur, B. *et al.* Improving pathological structure segmentation via transfer learning across diseases. In *Domain Adaptation and Representation Transfer and Medical Image Learning with Less Labels and Imperfect Data* 90–98 (Springer, 2019).

[57] Zhou, H.-Y., Oliver, A., Wu, J. & Zheng, Y. Training strategies, models and datasets. When semi-supervised learning meets transfer learning. *CoRR* (2018).

[58] Mehrtash A (2019). Automatic needle segmentation and localization in MRI With 3-D convolutional neural networks: Application to MRI-targeted prostate biopsy. IEEE Trans. Med. Imaging.

[59] De Vente C, Vos P, Hosseinzadeh M, Pluim J, Veta M (2020). Deep learning regression for prostate cancer detection and grading in bi-parametric MRI. IEEE Trans. Biomed. Eng..

[60] Kingma, D. P. & Ba, J. L. Adam: A method for stochastic optimization. In *Int. Conf, Learning Representations (ICLR)* (2015).

[61] Mehrtash A, Wells WM, Tempany CM, Abolmaesumi P, Kapur T (2020). Confidence calibration and predictive uncertainty estimation for deep medical image segmentation. IEEE Trans. Med. Imaging.

[62] Fedorov A (2012). 3D Slicer as an image computing platform for the quantitative imaging network. Magn. Reson. Imaging.

[63] Litjens, G., Futterer, J. & Huisman, H. Data from prostate-3T. *Cancer Imaging Arch.*10.7937/K9/TCIA.2015.QJTV5IL5 (2015).

[64] Bloch, N. *et al.* NCI-ISBI 2013 challenge: automated segmentation of prostate structures. *The Cancer Imaging Archive* (2015).

[65] Clark K (2013). The cancer imaging archive (TCIA): maintaining and operating a public information repository. J. Digit. Imaging.

[66] Roth, H. R. *et al.* Deeporgan: Multi-level deep convolutional networks for automated pancreas segmentation. In *Med. Image Comput. Comput. Assist. Interv.* 556–564 (Springer, 2015).

[67] Roth, H. R. *et al.* Data from pancreas-ct. *The Cancer Imaging Archive* (2016).

[68] Xu Z (2016). Evaluation of six registration methods for the human abdomen on clinically acquired CT. IEEE Trans. Biomed. Eng..

[69] Landman, B. *et al.* Miccai multi-atlas labeling beyond the cranial vault-workshop and challenge. In *Proc. MICCAI: Multi-Atlas Labeling Beyond Cranial Vault-Workshop Challenge* (2015).

[70] Gibson, E. *et al.* Inter-site variability in prostate segmentation accuracy using deep learning. *International Conference on Medical Image Computing and Computer-Assisted Intervention* 506–514 (2018).

[71] Simpson, A. L. *et al.* A large annotated medical image dataset for the development and evaluation of segmentation algorithms. arXiv preprint arXiv:1902.09063 (2019).

[72] Gal, Y. & Ghahramani, Z. Dropout as a Bayesian approximation: Representing model uncertainty in deep learning. In *Int. Conf. Machine Learning (ICML)* 1050–1059 (2016).

[73] Huang, G. *et al.* Snapshot Ensembles: Train 1, get M for free. *CoRR* (2017).

