# Supplementary material for: Domain adaptation for segmentation of critical structures for prostate cancer therapy
Source: Sci Rep. 2021 Jun 1;11:11480. doi: 10.1038/s41598-021-90294-4 (PMC8169882; doi:10.1038/s41598-021-90294-4)
Supplement: Supplementary file 1 — Supplementary Information. [file 41598_2021_90294_MOESM1_ESM.zip › Segmentations Target Data/supplemental data info.pdf]

Domain Adaptation for Segmentation of Critical Structures for Prostate Cancer Therapy

Anneke Meyer, Alireza Mehrtash, Marko Rak, Oleksii Bashkanov, Bjoern Langbein, Alireza Ziaei, Adam S. Kibel, Clare M. Tempany, Christian Hansen, Junichi Tokuda

Supplementary Data

This supplemental data contains 3-class critical structures segmentations (prostate gland, EUS and NVB) created for 25 cases from the PROSTATE-3T dataset [1] available on the Cancer Imaging Archive [2]

The segmentations contain in part labels provided by NCI-ISBI 2013 Challenge [3] and NVB labels uploaded on the TCIA dataset site [1]. Further details on the creation of segmentation labels can be found in our paper in the subsection 'Target Data'.

[1] Litjens, Geert, Futterer, Jurgen, & Huisman, Henkjan. (2015). Data From Prostate-3T. The Cancer Imaging Archive. DOI: 10.7937/K9/TCIA.2015.QJTV5IL5

[2] Clark K, Vendt B, Smith K, Freymann J, Kirby J, Koppel P, Moore S, Phillips S, Maffitt D, Pringle M, Tarbox L, Prior F. (2013) The Cancer Imaging Archive (TCIA): Maintaining and Operating a Public Information Repository, Journal of Digital Imaging, Volume 26, Number 6 pp 1045-1057. DOI: 10.1007/s10278-013-9622-7

[3] Bloch N, Madabhushi A, Huisman H, Freymann J, Kirby J, Grauer M, Enquobahrie A, Jaffe C, Clarke L, Farahani K. (2015). NCI-ISBI 2013 Challenge: Automated Segmentation of Prostate Structures. The Cancer Imaging Archive. http://doi.org/10.7937/K9/TCIA.2015.zF0vlOPv
